# Chronic Inflammatory Demyelinating Polyneuropathy Following Natural Influenza A Infection in a Pediatric Patient: A Case Report and Literature Review

**DOI:** 10.1155/crnm/8840308

**Published:** 2025-05-05

**Authors:** Emily Grew, Garrett Gianneschi, Janet Elgallab

**Affiliations:** ^1^Department of Pediatrics, New York University-Langone, New York, New York, USA; ^2^Department of Neurology, Rutgers-New Jersey Medical School, Newark, New Jersey, USA

## Abstract

Chronic inflammatory demyelinating polyneuropathy (CIDP) following viral infections and influenza vaccination has been well documented. However, there have been no confirmed natural influenza A infections leading to development of CIDP. Therefore, we present the case of a 6-year-old male who developed CIDP following a confirmed influenza A infection. Initially presenting with typical flu-like symptoms, the patient experienced a gradual onset of gait instability and leg weakness approximately 1 month later. Despite initial improvement with intravenous immunoglobulin therapy following a diagnosis of Guillain–Barré syndrome, his symptoms relapsed, including lower extremity weakness, incontinence, and sensory loss. Electromyography confirmed a demyelinating polyneuropathy, leading to a diagnosis of CIDP.

## 1. Introduction and Literature Review

Chronic inflammatory demyelinating polyneuropathy (CIDP) is characterized by segmental and multifocal demyelination that may induce axonal loss with time [1]. Defined as acquired, immune-mediated, progressive, symmetric peripheral nervous system weakness that evolves over at least 4 weeks, CIDP is relatively uncommon in children and lacks a reliable biomarker [2]. Onset in children is often more precipitous (often presenting within 6 months vs. adults more often presenting more than 6 or 12 months after symptom onset), with gait abnormality being a more frequent presenting symptom [3]. Differential diagnoses in CIDP patients presenting with weakness, decreased DTRs, and ataxia, sometimes following a viral infection, may include Guillain–Barre syndrome (GBS) [4], acute cerebellitis [5], postinfectious cerebellar ataxia, acute demyelinating encephalomyelitis, multiple sclerosis, Lyme Disease, acute necrotizing encephalopathy, cerebellar abscess, and impaired cerebellar development secondary to congenital infection [6].

CIDP may present acutely, and 5% of GBS patients are later reclassified as A-CIDP [7]. A study of 43 pediatric patients diagnosed with CIDP found that 74.7% were initially diagnosed with GBS and only 11.6% with CIDP [4]. GBS incidence is 0.62 per 100,000 years in 0–9 year olds and 0.75 per 100,000 in 10–19 year olds [8]. Juvenile CIDP incidence is 0.23–1.26 per 100,000, with variability owing to different diagnostic criteria [9]. Multiple studies report 100% of pediatric CIDP patients presenting with decreased DTRs, 88%–100% having lower extremity weakness, and variable presence of sensory loss [4, 10]. However, CIDP has atypical presentation in upto 39% of the cases, including pure sensory or motor forms [6, 11] and asymmetric presentation [11]. Errors in electroencephalogram interpretation, CSF cytoalbuminologic dissociation, and subjective improvement on immunotherapy may lead to misdiagnosis [2]. Cytoalbuminologic dissociation is sensitive but not specific for CIDP, causing diagnostic uncertainty when differentiating from other diseases [2]. Other diagnostic criteria include nerve conduction study (NCS), which is strongly recommended but less useful in pediatric patients [2, 7], paranodal protein antibodies such as neurofascin [12], antiganglioside antibodies [13], and MRI nerve root enhancement. Prompt identification is important to initiate appropriate treatment using steroids and/or IVIG and to avoid additional unnecessary diagnostic workup. Response to IVIG may be a differentiating factor, while response to steroids is similar in some differential diagnoses [2]. Steroids are ineffective in GBS and are usually only used when CIDP is suspected, requiring a high degree of clinical suspicion [8]. Most CIDP patients initially treated only with IVIG, as may be done when GBS is suspected, were eventually started on steroids due to relapse or incomplete response [10]. Treatment options for refractory cases of CIDP include plasmapheresis with transient symptom improvement. Others included mycophenolate mofetil, rituximab, cyclophosphamide, azathioprine, and abatacept [14]. Despite a range of potential options, thus far, there is no clear treatment algorithm for CIDP in children [4].

Overall, preceding infection or vaccination are weak predictors for CIDP [9]. A total of 23%–48% of pediatric CIDP patients report preceding illness in the weeks before symptom onset, most often nonspecific upper respiratory infection (URI) symptoms [1, 3, 5, 10, 11, 15]. Viral infections documented to have preceded a diagnosis of GBS/AIDP or CIDP include *Campylobacter jejuni*, cytomegalovirus, Epstein–Barr virus, Varicella–Zoster virus, Hepatitis A, B, C, and E, SARS-CoV-2 (COVID-19), Zika virus, *Mycoplasma pneumoniae*, parvovirus, HIV-1, and GB virus C [16–22]. An adult study of 268 CIDP patients found 10.4% with preceding events, 9.3% of which were prior nonspecific respiratory or gastrointestinal (GI) infection, and 3 patients had preceding influenza vaccination [15]. Antecedent events were more common in younger patients with subacute onset of CIDP [15]. Other antecedent events in adults with CIDP include surgery, trauma, and cortisone injections [3]. Vaccination is reported in 11% of pediatric CIDP patients within 8 weeks of symptom onset, most often influenza vaccination, and vaccines may also trigger recurrence of prior CIDP symptoms [23].

Influenza has known possible neurologic complications, most often simple or complex febrile seizures [24–29]. Rarer complications include encephalitis/encephalopathy, meningitis, altered mental status, syncope, ataxia, opsoclonus, extremity paresis, acute disseminated encephalomyelitis, transverse myelitis, paralysis/GBS, acute necrotizing encephalopathy, and stroke [24–29]. Pre-existing neurologic disease is a risk factor for influenza-associated neurologic complications [25]. Of hospitalized pediatric patients with influenza, between 8.1%–11.8% had neurologic complications [24, 25, 28]. Influenza has also been linked as a trigger for non-neurologic conditions such as pneumonia, respiratory failure, myocarditis, cardiogenic shock, hemolytic uremic syndrome, secondary bacterial infections, multisystem inflammatory syndrome in children, rhabdomyolysis, and viral myositis [30–34].

CIDP has been described in two case reports of adult and pediatric patients after influenza vaccination, with diagnosis supported by antibody testing, NCS, MRI, and/or nerve biopsy, with response to IVIG and steroids [35, 36]. Symptom onset in pediatric patient was the same day as influenza vaccination and 18 days later for adult patient [35, 36]. Another CIDP mimic is cerebellar ataxia after a preceding infection, which may range from postinfectious ataxia to life-threatening acute cerebellitis [6]. Acute cerebellitis is most commonly reported after varicella but also reported from influenza, rotavirus, HHV7, EBV, and *M. pneumoniae* [6]. Acute postinfectious ataxia can be triggered by varicella, mumps, *M. pneumoniae*, EBV, and other nonspecific infections [6].

The pathogenesis of CIDP is currently attributed to molecular mimicry of foreign epitopes in infectious agents to self-epitopes in the peripheral nervous system as seen in GBS [37]. Autoimmune demyelination then occurs through the dual process of IgG4 autoantibody disrupting the neuronal axon-Schwann cell anchoring proteins, as well as macrophage phagocytosis of Schwann cell myelin [38]. Specifically, IgG4 autoantibodies target the neurofascin-155 and contactin-1 proteins in the paranodal region of the myelinated axon without triggering macrophage-induced demyelination. Macrophage-induced demyelination is activated by a separate complement cascade pathway [38].

Most reports of preceding infection prior to CIDP do not specify the cause of URI or GI symptoms, and CIDP has not been reported following influenza A infection thus far in the literature. We aim to describe a pediatric patient presenting with weakness, loss of DTRs, gait disturbance, and sensory loss 1 month after confirmed influenza A infection, eventually diagnosed with CIDP.

## 2. Case Report

**Figure 1:**
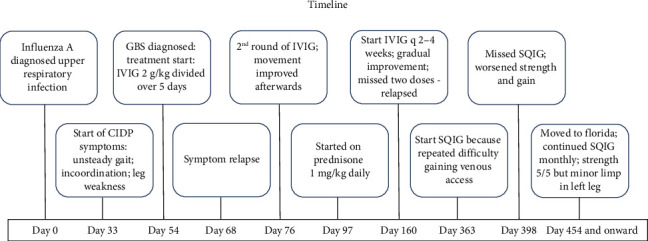
Timeline of events.

**Figure 2:**
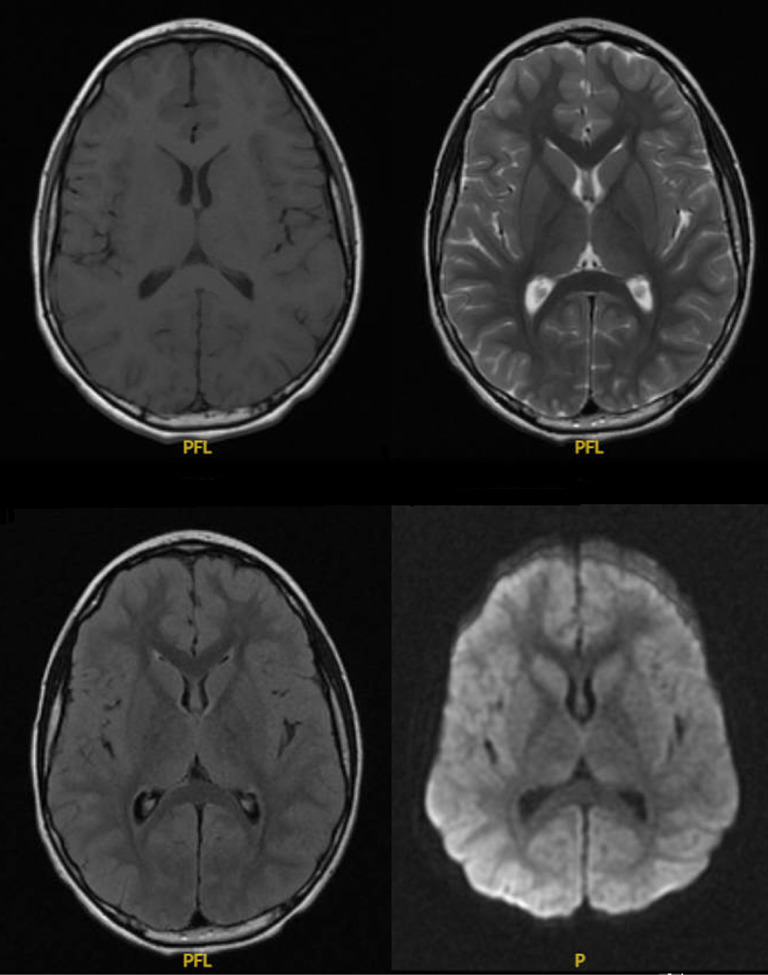
Clockwise from top left: Normal Axial T1, T2 FSE, Diffusion Weighted Imagine, and T2 FLAIR.

We present the case of a 6-year-old male who developed CIDP after an influenza A infection. A timeline of major events is shown in Figure 1. ZT originally presented to urgent care on Day 0 for 2 days of fever, cough, and body aches. He tested positive for influenza A, negative for influenza B, and negative for SARS-CoV-2. He was prescribed oseltamivir, amoxicillin, and pseudoephedrine and the symptoms resolved. The mother first noticed on Day 33 postinfluenza A infection, and ZT developed mild unsteady gait, incoordination, and leg weakness which steadily worsened. He presented to the emergency department on postinfection Day 54 for worsening symptoms. His exam showed diffuse 4/5 weakness, absent deep tendon reflexes (DTRs) in lower extremities, DTRs 1+ in upper extremities, decreased vibratory sense in bilateral toes, dysmetria of upper and lower extremities, dysdiadokokinesia of both hands, and unable to tandem walk. Given the physical HPI, exam findings, and recent viral illness, the differential diagnosis included GBS/acute inflammatory demyelinating polyneuropathy, postinfection ataxia, acute flaccid myelitis, transverse myelitis, myasthenia gravis, Lyme disease, postinfectious myositis or a space-occupying lesion within the spinal cord, vitamin deficiencies, and electrolyte/metabolic derangements. Comprehensive metabolic panel, complete blood count (CBC), erythrocyte sedimentation rate (ESR), C-reactive protein (CRP), and serum Lyme titers were all normal. Cerebrospinal fluid (CSF) analysis showed albuminocytologic dissociation with protein 128 mg/dL (ref: 15–45); CSF IgG 15.7 mg/dL (ref: < 4.3); and a CSF white blood cell count of 2 c/uL (Lymph% 100). CSF culture and CSF PCR were negative for all bacterial or viral CNS infections. Serum showed elevated ganglioside monosialic acid 2 IgG-IgM 63 (positive = 51–100) and elevated IgG, Quant 4265 mg/dL (ref: 538–1216). MRI brain was normal, as shown in Figure 2.

He was diagnosed with GBS and received intravenous immunoglobulin 2 g/kg divided over 5 days. His exam improved markedly; strength was globally 5/5, absent lower extremity DTRs, and 2+ DTRs in upper extremity, with improved gait and coordination. After discharge, there was a gradual, full relapse of symptoms over 1 month and he returned to the emergency department. In addition, he developed incontinence and sensory loss including decreased light touch and vibratory sense below the knees bilaterally. Electromyography and NCS showed an acquired demyelinating polyneuropathy most notable in bilateral median, ulnar, peroneal, tibial and sural nerves as shown in Table 1. References values for NCS were taken from the American Association of Neuromuscular and Electrodiagnostic Medicine and Ryan et al.'s study of pediatric NCS [39, 40]. Electromyography was normal.

He received formal diagnosis of CIDP based on abnormal NCS, albuminocytologic dissociation, elevated quantitative IgG, and elevated ganglioside monosialic acid 2 IgG-IgM (which suggested a demyelinating process) along with fulfilling criteria for CIDP based on European Academy of Neurology and Peripheral Nerve Society by fulfilling all of the following: [7]

• Progressive or relapsing, symmetric, proximal, and distal muscle weakness of upper and lower limbs, and sensory involvement of at least two limbs
• Developing over at least 8 weeks
• Absent or reduced tendon reflexes in all limbs.

He received a 1-year course of monthly immunoglobulin therapy and prednisone 1 mg/kg/day which was tapered because of weight gain and emotional lability, as shown in Figure 1. During that year, ZT's exam followed a relapsing and remitting course without a full return to baseline functioning during remitting periods with lower extremity weakness and abnormal gait as the most persistent and concerning issues. The episodes of remission did not consistently coincide with monthly administration of immunoglobulin therapy. Upon last follow-up at 1.5 years after initial presentation, he had significant improvement and was continued on monthly IVIG. His last recorded physical exam showed continuing motor deficit was hip flexion 5- out of 5 bilaterally, a minor limp in the right leg, minor decreased vibration sense below the ankles, and absent deep tendon reflexes in the knees and ankles. Throughout the course, his negative inspiratory force and vital capacity remained adequate, and he never required respiratory support. The family then moved away.

## 3. Discussion

This case report documents the presentation of CIDP following a natural influenza A infection in a pediatric patient. While the scientific literature has reported preceding infections in CIDP cases, the specific association between natural influenza A infection and CIDP remained unreported. Therefore, this case report helps to substantiate the widely held (yet undocumented) belief that CIDP can be caused by an influenza A infection.

This case demonstrates a typical presentation and clinical course of CIDP. His relapsing course, despite treatment, illustrates the unpredictable nature of CIDP in children and the difficulties in maintaining long-term remission. Notably, the patient's persistent weakness, absent deep tendon reflexes, and sensory deficits suggest that early intervention may not always prevent permanent neurological sequelae. Comparing patient ZT prognosis with larger pediatric cohorts, a pooled patient analysis of 209 pediatric patients showed 40.8% achieved remission (treatment-free stable condition for at least 1 year after treatment), while two smaller studies showed complete remission (no deficits) in 42.8%–51.4% and partial remission (stable with some residual deficits) in 10.8%–57.2% [41–43].

This case also brings to light the need for a more standardized treatment approach in pediatric CIDP, particularly in cases where symptoms fluctuate despite immunotherapy. While IVIG and steroids remain the first-line treatments, their long-term efficacy and the potential need for adjunctive therapies in refractory cases remain areas of active investigation. Alternative treatments such as plasmapheresis, mycophenolate mofetil, or rituximab may be considered in patients with persistent or relapsing symptoms.

Given the patient's improvement after receiving immunotherapy, but incomplete recovery, it is evident that a more comprehensive understanding of CIDP's pathophysiology, especially in the pediatric population, is needed. In addition, the impact of postinfectious immune responses and the role of antecedent infections, like influenza A, in triggering autoimmune neuropathies warrant further study.

In conclusion, this case report acts, in small part, to validate what the medical community has long suspected that a natural influenza A infection can contribute to the development of CIDP. The patient's fluctuating clinical course emphasizes the importance of close monitoring and individualized treatment plans. Future research should focus on optimizing therapeutic strategies and identifying biomarkers that could predict treatment response and long-term outcomes in pediatric CIDP patients.

## Figures and Tables

**Table 1:** Motor and sensory nerve conduction study.

**Motor nerve conduction studies**			
	**Distal latency (ms)**	**Amplitude (mV)**	**Conduction velocity (m/s)**

L median N.–APB	Ref: < 4.6	Ref: > 5.9	Ref: > 49
Wrist	5.99!	7.0	
Elbow	12.81!	3.5!	20.5!
R median N.–APB			
Wrist	5.10!	4.9!	
Elbow	13.33!	2.3!	17.0!
L ulnar N.–ADM	Ref: < 3.7	Ref: > 7.9	Below elbow ref: > 52 Above elbow ref: > 50
Wrist	4.64!	5.9!	
Below elbow	10.05!	4.1!	25.8!
Above elbow	14.79!	3.0!	14.8!
R ulnar N.–ADM			
Wrist	4.27!	6.4!	
Below elbow	10.99!	3.2!	20.8!
Above elbow	13.54!	2.5!	27.4!
L peroneal N.–EDB	Ref: < 6.5	Ref: > 2.6	Ankle to fib head ref: > 43 Across fib head ref: > 42
Ankle	10.21!	1.8!	
Fibular head	16.41!	1.6!	38.7!
Popliteal fossa	17.86!	1.6!	48.0
R peroneal N.–EDB			
Ankle	9.06!	2.3!	
Fibular head	15.99!	1.7!	34.6!
Popliteal fossa	19.22!	1.3!	21.7!
L tibial N.–AHB	Ref: < 6.1	Ref: > 5.8	Ref: > 44
Ankle	8.33!	3.6!	
Popliteal fossa	19.53!	2.1!	27.7!
L tibial N.–AHB			
Ankle	7.97!	4.1!	
Popliteal fossa	18.96!	2.2!	28.2!

**Sensory nerve conduction studies**			
		**Amplitude**	**Conduction velocity**

		Ref: > 13	Ref: > 55
Median, L (wrist), orthodromic		6.5!	50.9!
Median, R (wrist), orthodromic		3.4!	42.3!
Sural, bilateral		Absent!	Absent!

*Note:* Exclamation points designate abnormal values. Ref = reference value; L = left; R = right; N. = nerve; Fib = fibula.

Abbreviations: ADM = Abductor Digiti Minimi, AHB = Abductor Hallucis Brevis, APB = Abductor Pollicis Brevis, EDB = Extensor Digitorum Brevis.

## Data Availability

The data supporting the findings of this case report are available from the corresponding author upon reasonable request. Due to the nature of the case and concerns regarding patient confidentiality, some data may be restricted.
